# Gender, food systems, and climate in the 2025 Nutrition for Growth commitments

**DOI:** 10.3389/fnut.2026.1837675

**Published:** 2026-07-16

**Authors:** Abena Thomas-Mambwe, Elizabeth Margolis, Emily Custer, Valentine Granet, Cecilia Fabrizio

**Affiliations:** 1 Scaling Up Nutrition Movement Secretariat, Geneva, Switzerland; 2 World Vision International, Uxbridge, United Kingdom; 3 Stronger Foundations for Nutrition, Washington, DC, United States; 4 Global Alliance for Improved Nutrition, Geneva, Switzerland; 5 Micronutrient Forum, Washington, DC, United States

**Keywords:** gender and nutrition, gender policy analysis, Nutrition for Growth commitments, Nutrition for Growth Summit, food systems, climate change, climate adaptation and mitigation, nutrition policy analysis

## Abstract

There is a significant need for funders and policymakers to more systematically integrate gender and nutrition across sectors, especially within climate and food systems. In 2025, the Paris Nutrition for Growth (N4G) Summit included, for the first time, a dedicated thematic area on gender equality. This research report analyzes the integration of gender, food systems, and climate action within the 2025 N4G commitments. Of the 631 commitments analyzed, 69.7% were categorized as having “No indicated connection to gender.” Among those with a specific gender focus (177/631, 28%), the vast majority (83.1%) were “Targeted,” viewing women primarily as recipients. Within this group, 21.1% framed women’s nutrition only in relation to their reproductive roles and for the benefit of children. Only 2.5% of all commitments were “Inclusive,” recognizing women as agents of change, and 2.2% were “Transformative,” addressing the root causes of inequality. While nearly two-thirds of all commitments (63.5%) were related to food systems, only 13.2% addressed climate, indicating poor integration of climate with nutrition policymaking. Further, 65.8% of commitments related to food systems and/or climate did not incorporate gender. However, commitments categorized as “Inclusive” or “Transformative” demonstrated strong integration with food systems approaches. To address gender inequality across nutrition, food systems, and climate action, N4G commitments must move beyond targeting women as beneficiaries and instead focus on entry points identified by this review: community, male, and private sector engagement, multi-sectoral actions across food, education, health, economic, WASH, and social protection systems, strengthening policy environments, data systems, and advocacy.

## 1 Introduction

Gender inequality is both a cause and consequence of poor nutrition for women and girls (1). For the purposes of this paper, the terms gender and gender equality follow the common global health definition, referencing the social construct of gender, and power imbalances often experienced by women and girls, as well as other marginalized or intersectional groups (2). Gender inequality in nutrition is persistent with women facing higher rates of malnutrition than men: in 2024, 63.5 million more women experienced food insecurity than men (3). Globally, in 2021, 31.2% of women ages 15–49 had anemia compared with 17.5% of men, increasing the risk of cognitive and motor impairments, fatigue, and reduced productivity (4). Malnutrition in pregnant women and adolescent girls raises the risk of maternal mortality, premature births, low-birthweight infants, and intergenerational cycles of malnutrition, further entrenching gender gaps and resulting in annual losses in productivity and potential of approximately US$1.6 trillion (5). Yet quality nutrition for all is a fundamental right and a powerful catalyst for empowerment and economic growth, offering significant 23:1 returns on investment (6, 7). To realize these returns, women must be recognized as agents of change, not only as recipients of targeted interventions. Policymakers must address harmful social norms and other context-specific barriers to women’s and girls’ nutrition to resolve root causes of inequality, accelerate progress, and achieve ambitions for improved nutrition, health, and prosperity.

Gender norms that influence women’s food intake, domestic and caregiving responsibilities, and broader policy environments significantly hamper their access to nutritious food, financial resources, and health services for themselves and their children. While harmful social norms impacting women’s nutrition are context-specific, common examples include women eating last and least within households, preferential allocation of higher prestige, more nutritious foods to men, food taboos restricting women’s consumption of nutritious foods, often during pregnancy and breastfeeding, and ideas impacting body image (8). These vulnerabilities are compounded by intersectional factors, including those considered in this review: race, ethnicity, class, sexual orientation, ability, socioeconomic status, religion, indigeneity, and refugee status.

In October 2023, the Committee on World Food Security endorsed the *Voluntary Guidelines on Gender Equality and Women’s and Girls’ Empowerment in the context of Food Security and Nutrition*, recognizing that gender disparities and discrimination in food security still persist globally despite progress and global commitments (9). Therefore, further investment is needed to accelerate dissemination and implementation of guidance for gender equality in food security and nutrition policy and programming (10).

The Nutrition for Growth Summit is a global pledging moment to drive greater action toward ending malnutrition. Every four years, stakeholders including governments, civil society, donors, the private sector, and academia make commitments to improve nutrition (11). The March 2025 Paris N4G Summit provided a key opportunity to mainstream gender across sectors that impact nutrition, including food systems and climate. For the first time, the Summit encouraged commitment-makers to address gender through a dedicated thematic area on nutrition and gender equality. Given the fundamental importance of maternal nutrition to the health, development, and productivity of future generations, women’s and girls’ nutrition is often viewed solely within the reproductive role, a narrow framing that overlooks women’s intrinsic rights and well-being. This review analyzes the commitments made at Paris N4G to identify how commitment-makers integrated gender equality and intersectional factors across food systems and climate action.

## 2 Method

The objectives of this review were threefold:

1. Identify the extent to which gender and equity were integrated within 2025 N4G commitments,
2. Identify opportunities and entry points to drive stronger gender integration within food systems and climate action,
3. Recommend gender-responsive and transformative actions for N4G commitment implementation and accountability moving forward.

The N4G commitments were analyzed using a content analysis approach. First, reviewers developed an analytical framework presented below to define categories for each sector, using established definitions (Table 1). This analysis defined gender as a social construct informing the experiences of women and girls of social norms, expectations, and power relations (12). Intersectionality was defined as examining the various identities, relationships, and social factors individuals hold that can create compounded experiences of discrimination and privilege (13). This analysis determined the extent to which equity was reflected in commitments based on whether any intersectional identities identified by the Nutrition Accountability Framework (NAF)^1^ were mentioned.

**Table 1:** Analytical framework.

Sector	Sub-categories	Definitions
Gender	No indicated connection to gender Gender non-specific Targeted Inclusive Transformative	Based on the Paris N4G Recommendations for Developing Commitments on Nutrition and Gender Equality: Call to Action Targeted: Directly addressing the specific nutritional needs of women and girls. Inclusive: Engaging women and girls in the decision-making process (design and/or implementation of interventions, programs or commitments). Transformative: Working to break down systemic and structural barriers and increase women’s and girls’ agency, access, and control over resources that influence their nutrition outcomes. Gender (non-specific): Any reference to ‘gender’ or ministries related to gender without indication of specific action.
Food systems	No indicated connection to food systems Food systems non-specific Production Supply chains Food environments Consumer behavior Other	Aligned to Committee on World Food Security’s Food Systems Framework
Climate	No indicated connection to climate action Climate change non-specific Mitigation Adaptation	As defined by the Intergovernmental Panel on Climate Change
Equity	No indicated consideration of intersectional factors Race Ethnicity Class Sexual orientation Ability Socioeconomic status Religion Indigenous population Refugee status Other	As defined and determined by the NAF N4G Commitment Registration Guidance

Each commitment description, as entered into NAF, was reviewed and categorized individually according to the analytical framework definitions. Commitments were first categorized under “Food Systems” or “Climate,” and then by the level of “Gender” integration and inclusion of “Equity” within the described actions. The “Food Systems,” “Climate,” and “Equity” categories allowed multiple selections, while “Gender” was limited to a single selection based on the highest level of action indicated in the commitment description (with “No indicated connection” being lowest and “Transformative” being highest). For all sectors, if either “No indicated connection” or “non-specific” was selected, no additional categories could be selected.

Each commitment was independently reviewed and categorized by two reviewers. Discrepancies in categorization were resolved through discussion and consensus. Final categorizations were aggregated for analysis using Google Sheets and visualized in Looker Studio. Common themes and examples were also extracted.

The analysis was limited to the minimal details provided in N4G commitment descriptions and did not include supporting policies or documentation, as these were not consistently available. Analysis of other sectors, including health, education, water, sanitation, and hygiene (WASH), and social protection, was also beyond the scope of this research. Due to these limitations, the review did not use the standard Gender Continuum labels of “Unaware (Blind),” “Sensitive,” “Responsive,” and “Transformative” (14). Instead, the degree of gender integration was defined as stated above. These labels can be layered onto the Gender Continuum, depending on specific contexts and social and political environments.

A total of 634 commitments from the 2025 Paris N4G Summit were qualitatively reviewed, as registered in the NAF (15). Initially, in June 2025, the review team analyzed 582 commitments; this analysis was updated in December 2025 with an additional 52 commitments. Three commitments were excluded due to incomplete or missing descriptions; therefore, this analysis is based on 631 N4G commitments made in 2025.

## 3 Results

### 3.1 Key findings

**Figure 1:**
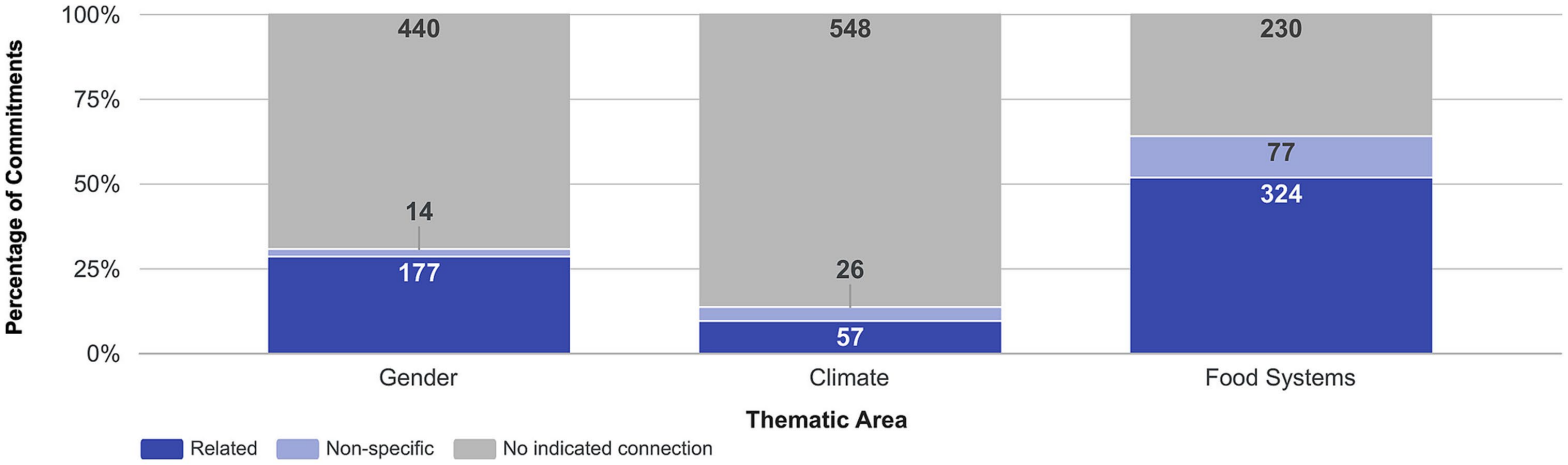
Most of the 631 commitments had no indicated connection to gender or climate.

**Figure 2:**
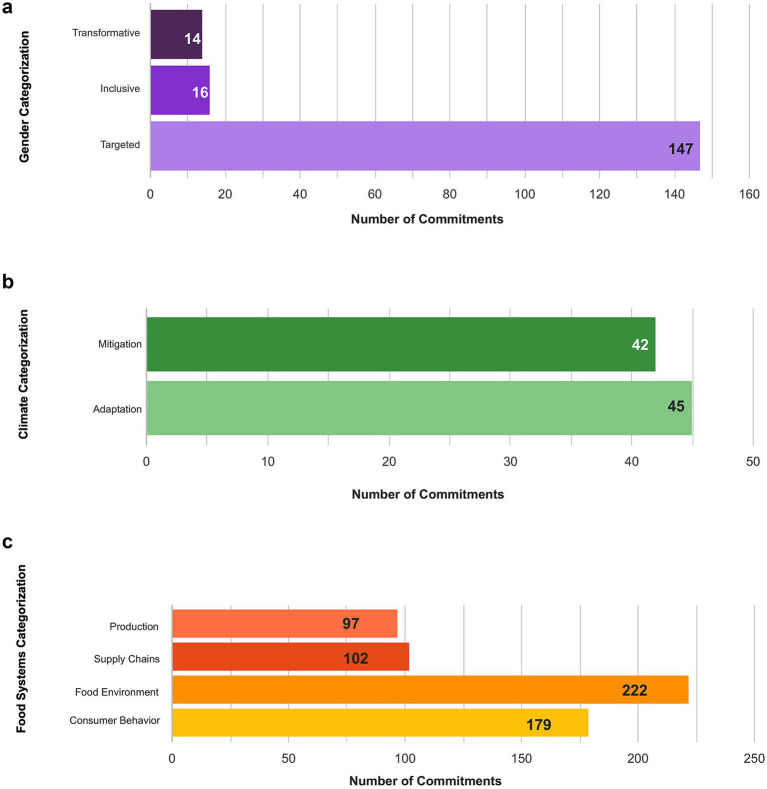
Sector-related commitments that address specific domains. **(a)** Over 80% of the 177 gender-related commitments were “Targeted” and only 8% were “Transformative”. **(b)** Most climate-related commitments (*n* = 57) included both “Mitigation” and “Adaptation” actions. **(c)** Most food systems-related commitments (*n* = 324) included at least one action related to “Food environment”. * Each commitment analyzed across each sector (gender, climate, food systems and equity). ** Categorization of commitments by gender was limited to a single-selection of domains. The degree of gender integration was based on the Paris N4G 2025 Recommendations for Developing Commitments on Nutrition and Gender Equality: Call to Action. ***Categorization of commitments by climate, food systems and equity allowed by multiple selection of domains, as defined by the analytical framework (see Methodology),

The majority – 69.7% (440/631) – of the Paris N4G commitments had “No indicated connection to gender” (Figure 1). Commitments including gender-related actions were largely “Targeted,” comprising 23.3% (147/631) of all commitments and 83.1% (147/177) of those related specifically to gender. Only 2.5% (16/631) of all commitments were “Inclusive,” and 2.2% (14/631) were “Transformative” (Figure 2a).

Only 9% (57/631) of all commitments included explicit actions tied to climate “Mitigation,” “Adaptation,” or both. An additional 4% (26/631) were categorized as “Climate non-specific” (Figure 1). Of the 57 climate-related commitments, 42 included at least one action related to “Mitigation” and 45 included at least one “Adaptation” action (Figure 2b), demonstrating prioritization of both mitigation and adaptation within climate-related commitments. The low representation of climate action within nutrition commitments limited this analysis” discussion of climate integration.

Roughly half (51.4%; 324/631) of all commitments included specific actions related to food systems, while an additional 12.2% (77/631) made general references to food or agriculture (“Food systems non-specific”) (Figure 1). Among food systems-related commitments, 29.9% (97/324) included actions related to “Production,” which may indicate a positive shift towards policymakers prioritizing production of nutrient-dense foods. Additionally, 31.5% (102/324) included actions related to “Supply chains,” 68.5% (222/324) to the “Food environment,” and 55.3% (179/324) to “Consumer behavior” (Figure 2c). Given the high-level nature of political commitments made at N4G, it is not surprising that nearly 70% addressed policy environments within food systems.

**Figure 3:**
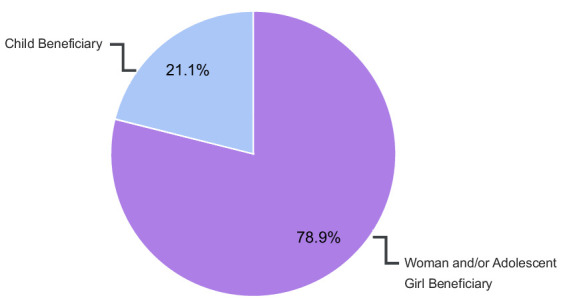
One in five commitments targeting women (31/147) focused on children as the ultimate beneficiary.

Within the commitments categorized as “Targeted,” 21.1% (31/147) placed children, particularly those under 5, as the primary beneficiaries, with limited or no consideration of women’s or girls’ nutrition status independent of motherhood or caregiving (Figure 3). This reflects an ongoing constraint in which women’s and girls’ nutritional needs are understood primarily or solely through their reproductive roles and as a means to address child malnutrition (1, 16). These commitments also did not consider harmful gender norms and barriers or women’s and girls’ active inclusion, participation, agency, or empowerment.

Only 17% (106/631) of all commitments considered dimensions of equity and intersectionality, identifying other social determinants or factors that intersect with gender. Most of these references focused on age (analyzed within the “other” category), particularly adolescents and youth, who are common targets of nutrition and economic development agendas. Despite frequent targeting in commitments, there remains a need to deepen transformative actions for adolescents, especially through adolescent-responsive programming and policies. Other intersectional factors referenced included socioeconomic status, refugee status, Indigenous populations, ethnicity, and ability. These references were rare, each appearing fewer than five times across all 631 commitments.

It is important to note discrepancies between self-reported tagging by commitment-makers during the submission process, and categorizations assigned by external reviewers in this analysis. There were 211 commitments self-reported within the gender theme, while 191 commitments were categorized by reviewers as “Gender non-specific” or related to gender (“Targeted,” “Inclusive,” or “Transformative”). Overall, 63.2% of commitments were categorized consistently (either related to gender or not) by both commitment-maker and reviewers, while 19.8% were self-reported as related to gender but categorized as “No indicated connection to gender” by reviewers. Further, 17% were categorized as “Gender non-specific” or related by reviewers but not identified by commitment-makers. This could in part be due to the review’s scope excluding background policy and documentation, the commitment submission format facilitating thematic selection, or the non-technical background of staff registering commitments.

For food systems and climate, 58.8% of commitments were categorized similarly by both commitment-makers and reviewers, while 32% were categorized as related to food systems and/or climate by reviewers but not identified as related by commitment-makers. Many commitments were self-reported as related to health but not food systems, reflecting limited recognition of the co-benefits of “health” interventions in shaping food systems (e.g., infant and young child feeding, breastfeeding counseling, and regulatory frameworks). Reviewers in this analysis tagged these areas within food systems as “Consumer behavior.”

### 3.2 Inclusive and transformative commitments

Entry points for meaningful policy change can be seen in the small subset (30/631) of commitments labeled “Inclusive” or “Transformative.” Within food systems, these 30 commitments ensured allocation of resources to female and youth farmers and producers, pledged to increase the proportion of female and youth employees in food processing and production, and acknowledged women’s roles in driving community-led solutions. Three commitments addressed gender gaps in data collection and information systems and promoted increased data-sharing on gender disparities.

Six “Transformative” commitments targeted opportunity structures [institutions, laws, and norms affecting women’s and girls’ agency and access to resources (17)] and addressed gendered barriers by supporting women’s choice to breastfeed and including extended paid leave and flexible hours. Three commitments increased women’s access to and control over resources, including loans, savings groups, and social protection schemes. Two commitments integrated education, healthy diets, and access to adolescent-friendly reproductive health services. Two others included male engagement through community groups to strengthen nutrition outcomes. One commitment launched a national campaign to reduce school dropout among girls, including gender-sensitive health and nutrition programming in schools.

Cross-analysis of food systems, climate action, and gender showed only modest integration; among climate- and food systems–related commitments, two-thirds (65.8%; 267/406) had “No indicated connection to gender.” This indicates a gap in recognizing the role of women and girls in addressing climate change and the transition toward resilient and sustainable food systems for improved nutrition.

Cross-analysis of the 30 “Inclusive” and “Transformative” commitments with food systems and climate show that gender linkages to food systems were far more common than linkages to climate; 27 of 30 were related to food systems, while only 10 were related to climate. Commitments with a meaningful understanding of the relationship between nutrition and gender inequality were also well integrated with food systems and, to a lesser extent, climate, demonstrating promising practices for integrated approaches to achieve multi-sectoral outcomes.

## 4 Discussion

### 4.1 Deepening commitments to integrate gender equality into nutrition policymaking

Despite the introduction of a Gender Thematic Area in the 2025 N4G process, nearly 70% of commitments failed to reference gender, with the vast majority of mentions being “Targeted” rather than “Inclusive” or “Transformative”. While it is encouraging to see growing awareness of the connection between gender and nutrition, merely referencing gender in nutrition commitments will not adequately address the root causes of malnutrition among women and girls. Findings related to equity reinforce this conclusion: while vulnerable groups are sometimes targeted, they are rarely included as agents of change, and the root causes of inequality remain unaddressed.

The fact that 21% of “Targeted” commitments ultimately focused on children, rather than women, indicates that policymakers continue to view women’s nutrition primarily as a means to improve outcomes for future generations, rather than for women themselves. Nutrition outcomes benefiting children, such as reductions in low birthweight and increases in exclusive breastfeeding, should also be framed around women-centric goals, including reducing maternal anemia, lowering breastfeeding mothers’ risk of ovarian and breast cancer (18, 19), and enhancing women’s overall well-being, productivity, and income generation.

Before being established as a dedicated thematic area in 2025, gender was even less visible in commitments from the previous two N4G summits. Analysis of the 2021 N4G Tokyo Compact by one of the authors found that only four Member States explicitly referenced gender in their commitments, an increase from the inaugural 2013 London Summit (20). This review of the 2025 commitments found that 53 Member States referenced gender in some way. While these analyses indicate growing consideration of gender in nutrition policy, there remains a significant gap in the meaningful integration of gender.

More broadly, women’s and girls’ well-being, needs, preferences, responsibilities, interests, and benefits should be recognized in their own right, rather than conceptualized primarily through a reproductive and caregiving lens. Prioritizing women’s and girls’ nutrition for its own sake is essential to breaking cycles of poverty and advancing gender equality in nutrition outcomes.

Against the backdrop of growing conflict and climate crises and decreasing global funding, policymakers must think more systematically about integrating nutrition to address multiple interconnected goals and deliver better results across all systems (see the Global Compact for Nutrition Integration) (21).

### 4.2 Recommendations for commitment-makers

1. Continue to increase the focus on linkages between gender and nutrition, as well as food systems, climate action, and other sectors (e.g., health, social protection, WASH, and education). This may include highlighting these linkages more explicitly in policies, and ensuring funding and collaborations reflect and support these connections.
2. Recognize women and girls as agents of change within nutrition policy, rather than primarily framing them through reproductive roles.
3. Engage men and community leaders to shift social norms harming women’s nutrition and gender equality.
4. Conduct analyses of context-specific gendered social norms, barriers, and intersectional factors, to tailor commitments, policy, and programs that transform, empower, and build resilience among the most vulnerable.
5. Develop and use gender-responsive data collection methods, encourage data sharing, and integrate sex-disaggregation into data systems to support identification of gendered nutrition trends and measure and accelerate progress toward global targets.
6. Leverage the entry points identified in this review to better integrate gender within the implementation of commitments moving forward.

### 4.3 Entry points for gender from inclusive and transformative commitments

This review seeks to equip policymakers to transform women’s nutrition by addressing key gaps and opportunities: transforming harmful gender norms and positioning women as agents of change whose nutrition is worthy of investment. Based on existing literature and the 30 “Inclusive” and “Transformative” commitments, this review identified the following entry points:

- Full participation in leadership and decision-making: promote equitable engagement of women, youth, and other marginalized groups within food systems transformation and nutrition, including in policymaking, service delivery, community engagement, and household decision-making.
- Economic empowerment in food systems: support women’s income generation by enhancing access to financial services and control over resources, prioritizing lending and microfinance for female entrepreneurs. Expand women’s access to agricultural resources, including land, seeds, inputs, information, technology, supply chains, and markets, and provide training in climate-smart agriculture (22). Enable women’s participation in and stimulate purchasing demand from female cooperatives.
- Community engagement for nutrition education: engage men, boys, and community leaders in social and behavior change efforts targeting harmful gender norms and food taboos. Train female teachers, agricultural extension workers, and healthcare workers to deliver gender-sensitive nutrition and health education in schools and communities. Promote peer-to-peer learning through mechanisms such as mother support groups and community daycare centers. Expand school feeding initiatives to create healthier school environments and promote gender-responsive health and nutrition education.
- Health: integrate delivery of iron and folic acid supplementation with sexual and reproductive health services. Strengthen prenatal nutrition services by scaling up delivery of the minimum package for maternal and adolescent nutrition. Train health workers to support micronutrient supplementation, exclusive breastfeeding, and diverse diets.
- Policy environment for breastfeeding: adopt supportive labor policies, including paid parental leave and baby-friendly workplaces (23). Establish breastfeeding corners, workplace daycare centers, and supportive public infrastructure aligned with the World Health Organization’s Baby-Friendly Hospital Initiative (24). Monitor and enforce compliance with the International Code of Breast-milk Substitutes (BMS Code) to prevent inappropriate promotion of infant formula.
- Social protection: implement and strengthen social safety net programs targeting women, youth, and other marginalized groups. Direct cash transfers toward improving nutrition outcomes, food security, resilience, and quality of life.
- WASH: promote investments that simultaneously address disease outbreaks and climate change while reaching the most vulnerable with equitable access to WASH services. Reduce the burden of water collection on women and girls and improve menstrual hygiene management.
- Multi-sectoral nutrition plans: develop and implement gender-responsive, multistakeholder nutrition action plans. Develop guidance to mainstream gender multi-sectorally, addressing access to and control over resources, as well as leadership and decision-making for women and girls. Elevate women’s and girls’ nutrition and empowerment as essential to achieving national nutrition targets.
- Data: include national and sub-national targets in national nutrition plans to reduce all forms of malnutrition among women and girls. Mainstream and harness sex-disaggregated and gender-disaggregated data to ensure nutrition investments benefit women and men equally; increase data-sharing on gender gaps to facilitate peer learning.
- Advocacy: prioritize advocacy efforts that address the root causes of harmful norms impacting women’s and girls’ nutrition. Engage local and international organizations, women’s groups, smallholder farmers, research entities, child rights organizations, and youth leaders to drive sustainable, community-led solutions.
- Private sector engagement: invest in food system infrastructure, including food processing and storage facilities, while ensuring equitable access for female producers. Strengthen private sector awareness of and compliance with relevant laws and regulations, including the BMS Code.

## 5 Conclusion

While the Paris N4G commitments offer a baseline for strengthening gender integration in nutrition-focused initiatives, this analysis indicates that substantial work remains to close the gender-nutrition gap. Commitments addressing gender too often position women primarily as beneficiaries within their reproductive roles, with significant gaps in gender-inclusive and gender-transformative actions. Furthermore, there is a notable lack of integration between gender, food systems, and climate across most commitments, despite the critical roles women play in these systems and the unique barriers they face.

To advance equity and nutrition outcomes for women and girls, particularly in an increasingly constrained funding environment, future policies and funding must cultivate and leverage women’s roles as agents of change and decision-makers. This requires moving beyond a narrow reproductive framing of women’s and girls’ nutritional needs and addressing the underlying social norms and systemic barriers limiting their full participation and empowerment within food systems and climate action.

A systems approach to gender analysis and integration is essential. This includes examining structural barriers, transforming unequal gender norms, and engaging men and community leaders to shift harmful social norms. By implementing these recommendations, commitment-makers can ensure future initiatives catalyze resilient and sustainable outcomes for the most nutritionally vulnerable populations.

## Data Availability

The original contributions presented in the study are included in the article/supplementary material, further inquiries can be directed to the corresponding author.

## Footnotes

1 The NAF is the formal entry portal for all N4G commitments.
